# Treatment of Pneumonitis, with Illustrative Cases

**Published:** 1881-07

**Authors:** P. O’Connell

**Affiliations:** Sioux City, Iowa


### ARTICLE III.

### TREATMENT OF PNEUMONITIS, WITH ILLUSTRATIVE CASES. By P. O'CONNELL, M.D., Sioux City, Iowa.

During the past four years the treatment of pneumonitis has been frequently discussed in current medical literature. Several able articles have appeared, from time to time, in the journals, advocating and detailing different and equally successful plans of treatment. The hope that others may try a method which is simple, inexpensive, and easily carried out, with the same gratifying result that I have had from it, prompts me to forward this contribution to the literature of the subject. Possibly the good result attained under very simple treatment may be a coincidence—may be only the natural history of pneumonia and not the result of the treatment employed. Although being *post hoc*, I consider it fairly *propter hoc*. Acute idiopathic lobar ("croupous?") pneumonitis is referred to in this connection.

The *antipyretic treatment*, in contradistinction to that usually followed by most practitioners in hospital and in private practice, consists essentially of the cold bath, large doses of quinine, and salicylic acid.

The *cold bath* is a very powerful means of abstracting heat from the body. The patient is kept in the bath during ten to twenty minutes or more, or until the temperature is reduced to about 100° F. The bath is always repeated under the personal supervision of the medical attendant, as often as the temperature mounts up to 103° F., or over. It requires frequent repetition, the reaction being often great and sudden, and the effect, therefore, but temporary. It certainly is difficult and troublesome to carry out, and is not seldom attended with some risks and unpleasant consequences. To patients and friends this plan seems not only heroic, which it really is, but positively appalling, and is consented to reluctantly. After fair trial it has not yielded very good results, and now has few advocates except among hospital physicians among English speaking peoples. For private practice it is practically useless.

*Quinine*, in some form, has many more advocates, and deservedly so. It is given by some in grain doses every hour, or five grains every four hours, or ten grains three times a day. Others give twenty to thirty grains morning and night, while a few give forty to fifty grains at once, and do not repeat the dose for twenty-four hours.

At Bellevue Hospital, New York, the cold bath was tried. The result was disappointing, and it was soon given up. Dr. James, of Frankfort, Kentucky, after a fair trial of the cold bath does not think very highly of it. In his opinion the result was not proportionate to the disturbance to the patient, nor to the risks, nor to the labor necessary to carry it out. With it the deaths amounted to seventeen per cent.; with quinine, in large doses, the death rate was twenty per cent.; salicylic acid proved a total failure in his hands. It is but just to say that the above figures were obtained by Dr. James during an outbreak of sewer gas pneumonia, when the mortality will necessarily be high under any and all forms of treatment.

Antimony, for its expectorant and diaphoretic effects, will be beneficial in some cases. Its sedative action on the heart proves very useful occasionally. In strong, vigorous patients I have found it answer well. Children under five years of age cannot, of course, safely take it except in rare cases.

Opium, especially when there is much pain, will be both useful and necessary. Diaphoretics and expectorants, to which may be added small doses of opium and sometimes antimonial wine in nauseating or non-nauseating doses, as occasion may require, will often prove a useful combination. Yet, out of a total of sixty-four cases treated by Dr. Thomas Barr, of Glasgow, Scotland, with antimony, opium, diaphoretics and expectorants, or with a combination of these agents, he had a mortality of *one in six* in private practice.

There is quite a unanimity of opinion as to the benefit of external applications to the chest, over the inflamed lobe or lobes, linseed poultices being in greatest favor. Moist warmth is both soothing and agreeable to the patient. Frequently it is all that is necessary to relieve the stitch-like pain. In my opinion it favors and hastens resolution. Mustard, turpentine, and even blisters, occasionally, may be required, but not until the consolidation of the pulmonary parenchyma tends to linger.

The treatment which I now practice is as follows: A piece of thick white flannel, loosely wrung out of hot water, is wrapped round the chest and covered with some material impervious to air and moisture, such as oiled silk, gutta percha tissue, or thin oil cloth. If only the lower lobe in one or in both lungs be inflamed, the flannel need not extend higher than the axillæ. But when an entire lung is involved, then the flannel must cover the entire thorax, apertures for the arms to pass through being cut in it. I deem it essential, and, therefore, always insist, that the flannel shall extend completely round the chest and overlap a little, at the ends, on the front of the thorax, whatever may be the extent of lung tissue involved. The outer air-tight covering should be a little larger, every way, than the flannel, so that the heat may not escape under the upper nor lower edge, nor at the sternum where the ends overlap. In all this there is nothing new. Dr. Flint speaks of it in his Practice of Medicine. Flannel heated and covered in this way retains its warmth quite as long as a poultice. I have, occasionally, used linseed poultices, but give the preference to the hot moist flannel, because it is much more cleanly and less troublesome ; it is easily renewed by dipping it again in hot water and loosely wringing it. Besides, few can be relied upon to properly and efficiently make a linseed poultice.

Then genuine* James' Fever Powder (*Pulvis Jacobi Verus*), one to five grains every two, three, or four hours, according to age, is prescribed. Five grains, to adults, is the maximum dose employed by me, while one grain can be given to a child under six months. The warmth of the flannel, aided by the James' Powder, soon induces and maintains gentle diaphoresis; the stitch-like pain is relieved; respiration becomes fuller and less hurried; cough grows softer and less hacking; expectoration becomes easy; the temperature steadily declines; the patient soon feels quite comfortable. If pain be very severe, a small hypodermic injection of morphia may be given to adults, although this will, I believe, be rarely required. In the case of children or adults, extract of opium or of belladonna, rubbed up with a little glycerin, may be painted on the skin over the painful part before applying the hot flannel ; or tincture of opium, of belladonna, or of aconite root may be sprinkled on the flannel, whenever the attending physician should judge such useful. Children under five years of age will need an emetic of ipecacuanha once or twice a day, if they do not clear the lungs sufficiently by acts of coughing.

---

* Pulvis antimonialis is not James' Powder. It is a worthless substitute for it. See the CHICAGO MEDICAL JOURNAL AND EXAMINER for August, 1879, for a paper by me on True James' Powder

During illness, a light but nutritious diet of good soup, chicken broth, beef tea, and milk with a little bread or crackers, is allowed. If craved, I see no objection to a little chicken, mutton chop, or beef steak broiled. I have permitted such and seen no ill effects result. My chief reliance, however, is placed on milk, which is allowed *ad libitum.* Infants at the breast must depend on the mother's milk. If there be any evidence of prostration or depression, tonics and stimulants are administered. These I rarely had to employ.

The fifteen cases, of which brief abstracts are given below, may be interesting for the following reasons: 1. The disease was typical in all. 2. The beginning of illness was accurately determined in each case. 3. The circumstances and hygienic surroundings were, to all intents and purposes, the same in all. 4. The season of the year (cold weather) was the same in all except three. 5. All the cases were in private practice, and were under my personal care and daily observation from the beginning to the end of illness. 6. Careful physical examination of the chest was practiced daily in each case. 7. The treatment in all was identical and as described above, and was faithfully carried out. 8. In all the disease ended in complete resolution.

The duration of the disease is computed from the outset (which, in the majority, coincided with the time the patient took to the bed) as accurately determined, as I said already, by personal physical exploration of the chest, to the time when *convalescence* was *well established.* In a few cases the nature of the illness was determined before the patient was compelled to keep the bed. Ten, or two-thirds of the cases were children up to twelve years of age, and among whom delirium at night, of an active character, was a prominent feature in about one-half. I append very short notes of each case.

CASE I. Michael M., aged 32, very recently suffered from syphilitic caries of the vomer ending in perforation, walked into my office. Felt chilly. *Lowest lobe of right lung* becoming consolidated. Had rust-colored expectoration. Was six days ill. Recovery complete.

CASE II. Mrs. C., aged 31, multipara, nursing. Was extremely peevish and could not be induced to keep quiet in bed.

To this I attribute the long duration of her illness. *Left lower lobe* involved; had malar blush; was eleven days ill. Recovery complete.

CASE III. Eddie C., aged 4, robust. Had chill. *Lowest lobe in each lung* involved. Was nine days ill. Recovery complete.

CASE IV. Mortimer S., aged 5, recovering from a severe impetigo. Malar blush well marked. *Lowest and middle lobes of right lung* involved. Was six and a half days ill. Recovery complete.

CASE V. Lily H., aged 8, a delicate child. Had chill; well marked malar blush. *Right lowest lobe involved.* Was six days ill. Recovery complete.

CASE VI. Mrs. W., aged 39, multipara (eleven children), very fat, flabby, a beer drinker, recovering from acute rheumatism which induced labor at end of eighth month, five days ago. *Entire right lung* involved. Had rust-colored expectoration; malar blush on right side only. Was seven days ill. Recovery complete.

CASE VII. Emma W., aged 7, delicate, syphilitic (acquired by kissing) for past three years. *Entire right lung and left lower lobe* involved. Had malar blush and herpes at angles of mouth. Was seven and a half days ill. Recovery complete.

CASE VIII. Jennie M., aged 12, very slow, dull child. Had well marked malar blush. *Entire right lung* involved. Was seven and a half days ill. Recovery complete.

CASE IV. Willie C., aged 23 months, robust. Had malar blush. *Right lower lobe* involved. Was six days ill. Recovery complete.

CASE X. James S., aged 6 months, strong, nursing. *Right lowest lobe* involved. Was six days ill. Recovery complete.

CASE XI. William C., aged 30½. Had malar blush and rust-colored expectoration. *Right lowest lobe* involved. Was three days ill. Recovery complete.

CASE XII. Freddie A., aged 14 months, strong. Had chill, malar blush, and rust-colored phlegm got up by emesis. Partially weaned. *Entire right lung* involved. Was six days ill. Recovery complete.

CASE XIII. James S., aged 17 months. Second attack with interval of eleven months health between. (See Case X.) *Left lower lobe* involved this time. Was three and a half days ill. Recovery complete.

CASE XIV. William C., aged 31. Second attack, with interval of six months health between. (See Case XI.) Had chill, malar blush, and rust-colored expectoration. *Entire right lung* involved this time. Was two and a half days ill. Recovery complete.

CASE XV. John G., aged 11, very strong lad. Had malar blush. *Entire right lung* involved. Was three days ill. Recovery complete.

The first of these fifteen cases was seen in November, 1877, and the last in February, 1880—a period of two and a half years. Four cases happened in the month of January, three in February, one in April, two in June, one in August, and four in November; all in the city of Chicago. Although cold may have played some part, possibly the greatest part, in the causation, still I attributed several of the cases to the action of sewer-gas, and have no reason now to alter that opinion. The average initial temperature in each case was 103.2° F.

The shortest mean duration of acute pneumonitis is twelve days according to the writers within my reach. The mean duration of my fifteen cases is six days, with a minimum of two and a half and a maximum of eleven days. From the beginning of illness until the patient was permitted to leave his bed and move about the house, and, in some cases, to go out of doors, was, on an average, seven days.

The relative frequency of the presence or absence of any one particular symptom of the disease can be seen at a glance. The extremely rapid manner in which a lobe or even an entire lung, as seen in cases XI, XIII, XIV, and XV, may become consolidated and just as quickly completely clear up again is interesting and remarkable.

